# From Anti-SARS-CoV-2 Immune Response to the Cytokine Storm via Molecular Mimicry

**DOI:** 10.3390/antib10040036

**Published:** 2021-09-24

**Authors:** Darja Kanduc

**Affiliations:** Department of Biosciences, Biotechnologies, and Biopharmaceutics, University of Bari, 70125 Bari, Italy; dkanduc@gmail.com; Tel.: +39-335-614-1668

**Keywords:** SARS-CoV-2 spike gp, cytokine storm, anti-inflammatory proteins, molecular mimicry, cross-reactivity, hyperinflammation, ferritinemia, macrophage activation syndrome, innate immunity, immunologic imprinting

## Abstract

The aim of this study was to investigate the role of molecular mimicry in the cytokine storms associated with severe acute respiratory syndrome coronavirus 2 (SARS-CoV-2). Human proteins endowed with anti-inflammatory activity were assembled and analyzed for peptide sharing with the SARS-CoV-2 spike glycoprotein (gp) using public databases. It was found that the SARS-CoV-2 spike gp shares numerous pentapeptides with anti-inflammatory proteins that, when altered, can lead to cytokine storms characterized by diverse disorders such as systemic multiorgan hyperinflammation, macrophage activation syndrome, ferritinemia, endothelial dysfunction, and acute respiratory syndrome. Immunologically, many shared peptides are part of experimentally validated epitopes and are also present in pathogens to which individuals may have been exposed following infections or vaccinal routes and of which the immune system has stored memory. Such an immunologic imprint might trigger powerful anamnestic secondary cross-reactive responses, thus explaining the raging of the cytokine storm that can occur following exposure to SARS-CoV-2. In conclusion, the results support molecular mimicry and the consequent cross-reactivity as a potential mechanism in SARS-CoV-2-induced cytokine storms, and highlight the role of immunological imprinting in determining high-affinity, high-avidity, autoimmune cross-reactions as a pathogenic sequela associated with anti-SARS-CoV-2 vaccines.

## 1. Introduction

Inflammation is a protective attempt by the organism to remove injurious stimuli, often represented by an infectious pathogen, and initiate healing processes. Ordinarily, inflammation is a stereotyped response and is considered as a mechanism of innate immunity [1]. However, as seen during the current SARS-Cov-2 pandemic, physiological inflammation may escalate into hyperinflammation [2] and associate with severe immunopathologies, from acute lung injury to thrombosis, vasculitis, and vasculopathy [3,4,5,6,7]. The molecular mechanisms that subvert a beneficial innate inflammatory response to SARS-CoV-2 into a pathological hyperinflammatory process are unknown.

Here, this study analyzes the degraded immunity associated with SARS-CoV-2 infection using the research paradigm developed since 1999 [8], according to which molecular mimicry between pathogen antigens and human proteins can trigger immune responses not only against the pathogen antigens, but also against the human host, with consequent cross-reactivity and autoimmune pathologies [9,10,11]. Accordingly, cross-reactive reactions between the SARS-CoV-2 spike gp and human surfactant seem to explain, for example, why SARS-CoV-2 so heavily attacks the respiratory system [12]. Such a cross-reactivity hypothesis appears to be substantiated by clinical data documenting that immunization with SARS-CoV antigens causes severe pneumonia [13], in this way also suggesting a dominant pathogenic role of anti-SARS-CoV antibodies in COVID-19. Moreover, importantly, SARS-CoV-2-reactive CD4^+^ T cells have been detected in ~40–60% of unexposed individuals, thus indicating cross-reactive T-cell recognition between circulating ‘common cold’ coronaviruses and SARS-CoV-2 [14], most possibly due to the phenomenon of ‘bystander activation’, whereby the infection may lead to activation of T cells that may in turn activate preprimed autoreactive T cells [15]. Furthermore, it has to be considered that comorbidities such as hypertension and diabetes induce chronic stress on endothelial cells with consequent abnormal expression of the molecules on their plasma membranes as an effect of post-translational modifications of intracellular proteins, including some heat-shock proteins. This can predispose cells and tissues to molecular mimicry phenomena that may occur during an infection [16].

Within this framework, in order to understand whether molecular mimicry and the consequent cross-reactivity might be capable of overturning the defensive innate immunity into a degenerated autoimmune self-attack, the present study explored human proteins related to anti-inflammatory activity for peptide sharing with the main antigen of SARS-CoV-2, i.e., the spike gp.

## 2. Materials and Methods

Peptide sharing between anti-inflammatory human proteins and the SARS-CoV-2 spike gp (NCBI, GenBank Protein Accession ID = QHD43416.1) was analyzed using pentapeptides as sequence probes since a peptide grouping formed by five amino acid (aa) residues defines a minimal immune determinant that can (1) induce highly specific antibodies, and (2) determine antigen–antibody specific interaction [17]. 

A library of 219 human proteins related to anti-inflammatory activity was assembled at random from the UniProtKB database (www.uniprot.org/, accessed on 1 June 2021) [18] using “anti-inflammatory” as a keyword. The 219 human proteins are listed in Supplementary Table S1.

The SARS-CoV-2 spike gp primary sequence was dissected into pentapeptides offset by one residue (that is, MFVFL, FVFLV, VFLVL, FLVLL, and so forth), and the resulting viral pentapeptides were analyzed for occurrences within the 219 human proteins that are related to anti-inflammatory activity. 

The human proteins involved in the peptide sharing (53 in total) were analyzed for functions/diseases using UniProtKB (www.uniprot.org/, accessed on 1 June 2021) [18], Pubmed, and OMIM (www.omim.org/, accessed on 1 June 2021) resources. 

Successively, using the Pir Peptide Match program (research.bioinformatics.udel.edu/peptidematch/index.jsp, accessed on 1 June 2021) [18], the shared peptides (namely, 59) were also investigated for occurrences in coronaviruses that were used as controls and are listed with NCBI:txid as follows: SARS-CoV (694009); Middle East respiratory syndrome (MERS)-CoV (1335626); human (H) CoV-229E (11137); HCoV-NL63 (277944).

Then, the immunological potential of the peptide sharing was analyzed by searching the Immune Epitope Database (IEDB, www.iedb.org/, accessed on 1 June 2021) [19] for immunoreactive epitopes hosting the shared pentapeptides. 

As a last step, pentapeptides common to SARS-CoV-2–derived epitopes and anti-inflammatory proteins were also controlled for occurrences in the bacterial pathogens *Bordetella pertussis* (257313), *Corynebacterium diphtheriae* (257309)*, Clostridium tetani* (212717), *Haemophilus influenzae* (71421), and *Neisseria meningitides* (122586).

## 3. Results 

### 3.1. Description of the Peptide Sharing between SARS-CoV-2 Spike Gp and Anti-Inflammatory Human Proteins

The peptide sharing between the SARS-CoV-2 spike gp and anti-inflammatory human proteins is quantitatively and qualitatively described in Table 1 and Table 2, respectively.

Table 1 shows that the SARS-CoV-2 spike gp vs. anti-inflammatory human protein peptide overlap amounts to 59 minimal immune determinants. When compared to the CoV controls, Table 1 shows that peptide sharing also occurs—to a lesser extent, but still a remarkable one—with SARS-CoV, which emerged in 2003 and, even if endowed with a lethal potential, could be controlled with public health measures [20]. None of the 59 pentapeptides are present in the additional CoV controls, i.e., the pathogenic MERS-CoV [21] or the scarcely pathogenic HCoV-OC43 and HCoV-229E CoVs that cause only mild symptoms [22]. In sum, Table 1 provides a quantitative description of a common, vast, and specific molecular platform that joins the SARS-CoV-2 spike gp and anti-inflammatory human proteins and, as well, supports the possibility of autoimmune cross-reactions between the SARS-CoV-2 antigen and the human host. 

Table 2 describes the distribution of the 59 SARS-CoV-2 spike gp pentapeptides among 53 anti-inflammatory human proteins.

At first glance, Table 2 reveals that the majority of the anti-inflammatory proteins involved in the peptide sharing are receptors implicated in cellular signaling pathways that regulate crucial processes such as proliferation, differentiation, apoptosis, and immune response. Space reasons do not allow a detailed one-by-one analysis of the 53 anti-inflammatory proteins listed in Table 2; hence, the discussion is here circumscribed to a few of the tabulated proteins.

#### 3.1.1. Adenosine Receptor A2b (AA2BR)

Table 2 begins with AA2BR, a seven-transmembrane receptor that binds adenosine, a regulator of innate immunity released into the extracellular (EC) space in response to metabolic stress and cell damage [23]. Following adenosine binding, AA2BR initiates the canonical cyclic adenosine monophosphate (cAMP) signaling pathway that, in succession, comprises activation of the heterotrimeric protein Gs, activation of adenylate cyclase (AC), production of cAMP, activation of protein kinase A (PKA), and phosphorylation of the cAMP responsive element-binding (CREB) protein that triggers gene transcription [24]. Immunologically, cAMP is a primary regulator of innate immune cell function [25]. High levels of cAMP reduce the production of proinflammatory mediators tumor necrosis factor (TNF)-α, interleukin (IL)-17, and interferon (IFN)-γ, whilst increase the production of anti-inflammatory factors such as IL-10 [26].

Consequently, hitting AA2BR equates to altering the cAMP levels and disintegrating the intracellular signaling network that regulates innate immune response. Simply put, cross-reactions hitting AA2BR would decrease cAMP production with consequent high levels of the proinflammatory TNF-α, IL-17, and IFN-γ. Clinically, pathological consequences would be suffered by almost all organs and, in particular, by the pulmonary and cardiovascular systems, since deficiency/alterations/improper functioning of AA2BR characterize acute lung injury and vasculopathies. In fact, AA2BR

- is expressed in all tested organs, including spleen, lung, colon, and kidney. Importantly, in all of these organs, the primary site of expression is the vasculature [27];
- is highly expressed on type II alveolar epithelial cells [28]. Of note, type II alveolar epithelial cells produce and secrete a pulmonary surfactant that reduces alveolar surface tension and prevents alveolus collapse during respiration [12,29]:
- has a potent anti-inflammatory role during acute lung injury [30,31];
- dampens inflammation particularly during tissue hypoxia [32];
- protects the colonic epithelial barrier during acute colitis [33];
- mediates different types of cardioprotection [34];
- when activated in the pre-adipocyte, inhibits adipogenesis [35];
- enhances the abundance of T regulatory cells (Tregs), a cell type critical in constraining inflammation [36,37]. Tregs constrain inflammation via multiple mechanisms depending on the tissue and nature of injury/inflammation (i.e., production of the anti-inflammatory cytokines IL-10, IL-35, and transforming growth factor β) [38,39].

In sum, cross-reactivity against AA2BR alone would be sufficient in itself to explain the acute respiratory syndrome and the systemic multiorgan hyperinflammation associated with SARS-CoV-2 infection.

#### 3.1.2. Adiponectin

Adiponectin is a versatile player of innate immunity [40]. This anti-inflammatory protein is an adipocyte-derived plasma protein [41] and a beneficial modulator for endothelial adhesion molecules [42] since it inhibits TNF-α-induced activation of the nuclear transcription factor κB (NF-κB) through a cAMP-dependent pathway [41]. Specifically, NF-κB is involved in the transcriptional regulation of vascular cell adhesion molecule-1, endothelial leukocyte adhesion molecule-1, and intracellular adhesion molecule-1. These adhesion molecules participate in the recruitment of leukocytes to inflammatory lesions and play a crucial role in monocyte adhesion to arterial endothelium [42,43,44]. The inhibitory effect of adiponectin and the concomitant cAMP accumulation are blocked by either AC inhibitors or PKA inhibitors [41].

Adiponectin stimulates expression of the potent anti-inflammatory IL-10 [45] and inhibits endothelial synthesis of inflammatory IL-8 [46,47]. Of note, hypo-adiponectinemia induces NLRP3 inflammasome activation [48,49,50]. Lastly, adiponectin paradoxically decreases in obesity [51].

On the whole, cross-reactions hitting adiponectin might lead to obesity, metabolic syndrome, diabetes, endothelial dysfunction, atherosclerosis, hypertension, and coronary artery disease [52].

#### 3.1.3. C163A Protein

C163A protein is a membrane-bound receptor, selectively expressed in macrophages. C163A acts as a hemoglobin (Hb) scavenger [53], and altered C163A is related to severe inflammatory syndromes. Indeed, under physiological conditions, senescent erythrocytes are phagocytosed and catabolized in macrophages [54], excepted for 10–20% of them that undergo intravascular hemolysis before being phagocytosed [55]. The release of Hb and its degradation products, heme and iron, in nonhematopoietic tissues can lead to oxidative stress because of the Fenton reaction [56], thus causing severe inflammatory pathologies such as stroke [56,57]. Physiologically, such inflammatory pathologies are prevented by the scavenger C163A protein that binds the complex Hb–haptoglobin (Hp) and mediates the uptake of the complex for catabolism in macrophages, thereby neutralizing and protecting from intravascular Hb/heme toxicity due to the Fe^++^-induced oxidative stress. 

Therefore, C163A deficiencies/alterations break down the protection against oxidative stress and cause intravascular accumulation of heme and iron with severe pathological outcomes. Indeed, the iron overload triggers the macrophage expression of C163A, while also triggering the expression of a disintegrin and metalloproteinase domain-containing protein 17 (ADAM17; alias: TNF-α-converting enzyme, TACE) [58,59]. The consequences can be devastating.

In fact, ADAM17 is a sheddase enzyme that cuts and sheds the EC domain of C163A [60], thus transforming the membrane-bound C163A receptor into the soluble C163A (sC163A) [61]. sC163A is still able to bind Hp–Hb complexes [62]; however, due to its being no longer bound to the macrophage membrane, it cannot mediate the uptake of the complex into the macrophages [63]. This creates a paroxysmal feedback loop in which the iron overload cannot be eliminated but instead further increases and further stimulates C163A and ADAM17 expression in macrophages, with ADAM17 nullifying the scavenger function of C163A and continuously producing sC163A. In parallel, the continuously increasing iron overload not only increases the oxidative stress but, in the attempt to neutralize the iron overload, also stimulates a continuous expression of ferritin with consequent hyper-ferritinemia [64]. To aggravate the pathological scenario, the increased ADAM17 sheddase can act on several transmembrane proteins with inflammatory properties such as TNF-α, the master-regulator of inflammatory cytokine production [65], thus unleashing powerful cytokine storms.

Such a succession of molecular events is supported by the fact that ADAM17 deficiency augments C163A-dependent apoptotic cell uptake into macrophages and the linked anti-inflammatory phenotype [66]. These events logically explain why high levels of sC163A are associated with the large spectrum of severe inflammatory disorders that are comprehensively grouped in the macrophage activation syndrome (MAS) and clinically include persistent high-grade fever, hepatosplenomegaly, lymphadenopathy, cytopenia, hemorrhagic manifestations, and a sepsis-like condition [67,68,69].

#### 3.1.4. ACs, CREB1, IL-10, IL27B, MY18A, and NLRC 

The immune dysregulated scenario that might originate from cross-reactivity against AA2BR, adiponectin, and C163A can be further disintegrated when one considers that targets of cross-reactions might include the following:

- ACs 4, 7, and 9, the alterations of which lead to cAMP decrease and consequent high levels of the proinflammatory TNF-α, IL-17, and IFN-γ [26];
- CREB1, a key transcription factor that, by inhibiting NF-κB activation, induces macrophage survival, and promotes the proliferation, survival, and regulation of T and B lymphocytes [70];
- IL-10, a major immune regulatory cytokine with profound anti-inflammatory functions [71];
- IL27B (aka EBI), which forms a heterodimer with IL12A known as IL-35 and a heterodimer with IL-27A known as IL-27 [72]. IL-35 and IL-27 are anti-inflammatory ILs. IL-35 promotes the proliferation and activation of Tregs and suppresses the function of T helper 17 cells and other inflammatory cells to inhibit immune responses [73], while IL-27 regulates innate immunity and controls microbial growth [74];
- MY18A, which is predominantly expressed in alveolar macrophages and plays an important role in pulmonary immunity by enhancing opsonization and clearance of pathogens and by modulating macrophage inflammatory responses [75];
- NLRC3 protein, which inhibits inflammation by disrupting NALP3 inflammasome assembly [76].

### 3.2. Immunogenic Potential and Immunologic Imprinting

#### 3.2.1. Immunogenic Potential of the Peptide Sharing

Obviously, cross-reactions hitting the above-described anti-inflammatory human proteins might lead to the cytokine storm and hyperinflammation syndromes that accompany exposure to SARS-CoV-2. This possibility is solidly supported by analyses of the immunological potential of the peptide sharing illustrated in Table 2. Indeed, Table 3 shows that, according to IEDB [19], almost all of the shared pentapeptides, with the exception of nine (namely, CGDST, FLVLL, GIAVE, LVLLP, NATRF, NDPFL, TGIAV, VAVLY, and VFLVL), occur and recur in 80 SARS-CoV-2 spike gp-derived epitopes that have been experimentally validated as immunoreactive, thus highlighting the concrete possibility of cross-reactions between the SARS-CoV-2 spike gp and anti-inflammatory human proteins.

#### 3.2.2. Immunologic Imprinting

Immunologically, it has to also be considered that, as already described [77,78,79,80,81], the extent and violence of the potential cross-reactions might considerably be intensified and heightened by phenomena of immunologic imprinting due to inter-pathogen peptide sharing. In fact, a distinctive property of the immune system is the memory for the immune determinants it has previously encountered so that, as a rule, the immune system reacts by recalling memory of the responses toward past infections rather than inducing ex novo responses toward the recent ones [82,83]. Such immunologic imprinting of immune determinants can be by and large common, in light of the massive and wide peptide overlap among pathogen proteomes [84,85].

Given this context, the 50 minimal immune determinants common to SARS-CoV-2 spike gp-derived epitopes and anti-inflammatory human proteins were analyzed for occurrences in bacterial pathogens such as *B. pertussis*, *C. diphtheriae, C. tetani*, *H. influenzae*, and *N. meningitides*, i.e., in pathogens to which, in general, individuals have already been exposed during their life due to infections or vaccination. The results reported in Table 4 illustrate an intense viral vs. bacterial peptide overlap capable of unchaining powerful and violent cross-reactions against the human host.

## 4. Discussion

In agreement with previous reports [77,78,79,80,81,84,85,86], the data described in Table 1 and Table 2 confirm that a vast peptide commonality joins viral and human proteins. Here, this study shows that the SARS-CoV-2 spike gp shares 59 minimal immune determinants with 53 anti-inflammatory human proteins that, consequently, may become susceptible to be targeted and altered/inactivated by autoimmune cross-reactions. The unexpected vastness and specificity of such peptide sharing can be mathematically appreciated when considering that the probability of two proteins sharing one pentapeptide on the basis of 20 aa is equal to 1 out of 20 raised to the power of 5, i.e., it is equal to 0.0000003125. Hence, the high level of the specific peptide sharing described in Table 1 and Table 2 opposes a casual peptide sharing phenomenon dictated by the pure mathematical laws of distribution and rather, once more, indicates an evolutionary process where viruses played specific roles in the origin of the eukaryotic cell [86].

Immunologically, a large part of the peptide sharing described in Table 2 is not only endowed with immunoreactivity (Table 3), but also has the potential to recall past infections and already targeted determinants (Table 4). Simply put, the inter-pathogen peptide sharing reported in Table 4 suggests that pre-existing immune responses to previously encountered pathogens—considering, in this case, *B. pertussis*, *C. tetani*, *C. diphtheriae*, *H. influenzae*, and/or *N. meningitides*—might be boosted following exposure to SARS-CoV-2. Therefore, the primary response to the virus might be transformed into a secondary (or even tertiary) response to the previously encountered pathogens of which the immune system has stored memory. This implies that the anamnestic and, by definition, extremely potent response against the bacterial immune determinants previously encountered may be predominant, whilst the immune response against the pathogen lastly encountered, i.e., SARS-CoV-2, may be weak or unsuccessful. As an additional consequence, the attack against the early sensitizing pathogens might fail because those early sensitizing pathogens are no longer present in the organism. In the end, a logical unavoidable result might be that the anamnestic, high-affinity, high-avidity, and extremely potent secondary immune response triggered by the lastly encountered pathogen, i.e., SARS-CoV-2, and addressed toward past infections can hit the only available targets, i.e., the immune determinants that, in this instance, are present in the human anti-inflammatory proteins. According to this sequence of events, molecular mimicry and immunological memory might explain the different pathological burden of the autoimmune responses—from zero or mild symptoms to severe, even lethal pathologies [80,81]—following exposure to SARS-CoV-2. In practice, the past history of the infections/vaccinations of each individual might be the main factor dictating the pathologic outcome.

Therefore, from a logical point of view, Table 3 and Table 4 powerfully support molecular mimicry and immunologic memory as the likely mechanistic links between SARS-CoV-2 infection and the immune dysregulation characterized by increased proinflammatory cytokines that affect COVID-19 patients. In this regard, the fact that peptide sharing specifically involves SARS-CoV-2, but not MERS-CoV (Table 1), is clinically noteworthy thus allowing the establishment of a causality relationship among shared peptides, potential cross-reactivity, and autoimmune pathological sequelae. Indeed, the lack of peptide commonalities between MERS-CoV and auto-inflammatory proteins described in Table 1 might explain the different immunological characteristics of the pathogenic SARS-CoV-2 and MERS-CoV [87].

## 5. Conclusions

The present report has special scientific-–clinical relevance in light of the consideration that, after decades of research [88,89,90,91,92,93], we still remain ignorant of the mechanism(s) via which respiratory infectious agents alter the immune defense of the human host by activating so-called ‘cytokine storms’ and cause physical occlusion of the pulmonary airways, with devastating pathologic sequelae in the systemic circulation. Indeed, scientifically, the present data indicate a molecular platform, i.e., peptide sharing, and a mechanism, i.e., molecular mimicry-induced cross-reactivity, to understand the etiology of the cytokine storm syndromes. Moreover, therapeutically, these data warn that antigens for vaccine formulation must be selected with some care. As repeatedly highlighted [9,12,17,80,81,86,94], the approach for specific, effective, and safe immunotherapies appears to reside in non-cross-reactive peptides.

## Figures and Tables

**Table 1:** Quantitative description of the pentapeptide sharing between CoV spike gps and anti-inflammatory human proteins.

Spike Gp from	Number of Shared Pentapeptides	Pentapeptide aa Sequence
SARS-CoV-2	59	AAAYY, AEIRA, AGAAA, AISSV, ALLAG, ASFST, AVRDP, CGDST, EKGIY, FLVLL, FNGLT, FSALE, FSQIL, GIAVE, GICAS, GTITS, GVLTE, IRAAE, IYQTS, KLQDV, KNLRE, KQLSS, KVEAE, LEILD, LGFIA, LIRAA, LLPLV, LPPLL, LVLLP, LVRDL, NATRF, NDPFL, NIIRG, NNTVY, NTFVS, PDKVF, PFFSN, PIGAG, QDSLS, QLSSN, QQFGR, QSIIA, RAAEI, SKVGG, SSNFG, SSVLH, TFVSG, TGIAV, TLADA, TLEIL, TLLAL, TMSLG, TNGVG, TSPDV, VAVLY, VFLHV, VFLVL, VLPPL, YSVLY
SARS-CoV	30	AEIRA, AISSV, CGDST, FNGLT, FSQIL, GICAS, IRAAE, IYQTS, KLQDV, KQLSS, KVEAE, LGFIA, LIRAA, LPPLL, NNTVY, NTFVS, PIGAG, QLSSN, QQFGR, RAAEI, SSNFG, TFVSG, TGIAV, TLADA, TMSLG, TSPDV, VAVLY, VFLHV, VLPPL, YSVLY
MERS-CoV	−	−
hCoV-229E	−	−
hCoV-NL63	−	−

**Table 2:** Peptide sharing between SARS-CoV-2 spike gp and anti-inflammatory human proteins.

Peptides ^a^	Anti-Inflammatory Human Proteins ^b^
**VLPPLL**	AA2BR. Adenosine receptor A2b.
ASFST, ALLAG	ADCY4. Adenylate cyclase type 4
LGFIA	ADCY7. Adenylate cyclase type 7
**KQLSSN**	ADCY9. Adenylate cyclase type 9
VLLPL	ADIPO. Adiponectin
SSNFG	C163A. Scavenger receptor cysteine-rich type 1 protein M130
**FLVLLP**	CALRL. Calcitonin gene-related peptide type 1 receptor
IYQTS	CREB1. Cyclic AMP-responsive element-binding protein 1
**LVLLPL**	CRFR1. Corticotropin-releasing factor receptor 1
IYQTS	DRD5. D(1B) dopamine receptor
VFLVL, TNGVG	FURIN. Furin
CGDST, TFVSG	GBB2. Guanine nucleotide-binding protein G(I)/G(S)/G(T) subunit β-2
TFVSG	GBB4. Guanine nucleotide-binding protein subunit β-4
NDPFL	GBGT2. Guanine nucleotide-binding protein G(I)/G(S)/G(O) subunit γ-T2
VFLHV	GLHA. Glycoprotein hormones α chain
QDSLS	GLP1R. Glucagon-like peptide 1 receptor
QSIIA	GNAI1. Guanine nucleotide-binding protein G(i) subunit α-1
KNLRE	GNAI2. Guanine nucleotide-binding protein G(i) subunit α-2
QSIIA	GNAI3. Guanine nucleotide-binding protein G(i) subunit α
**NTFVSG**	GNB5. Guanine nucleotide-binding protein subunit β-5
YSVLY, GICAS	GP176. G-protein coupled receptor 176
ALLAG	GPR25. Probable G-protein coupled receptor 25
LLPLV	GPR83. Probable G-protein coupled receptor 83
TLLAL	GPR84. G-protein coupled receptor 84
EKGIY	IL10. Interleukin-10
TMSLG	IL27B. Interleukin-27 subunit β (EBI)
VLLPL, KVEAE	ISK5. Serine protease inhibitor Kazal-type 5
QLSSN	KS6A5. Ribosomal protein S6 kinase α-5
VLPPL	LSHB. Lutropin subunit β
PFFSN	MC3R. Melanocortin receptor 3
GIAVE	MC5R. Melanocortin receptor 5
FNGLT	MXRA5. Matrix-remodeling-associated protein 5
SSVLH, NATRF, FSQIL, **LIRAAEI**	MY18A. Unconventional myosin-XVIIIa
FSALE	MYH9. Myosin-9
LVRDL	NFKB1. Nuclear factor NF-kappa-B p105 subunit
**TLEILD**, NIIRG, SKVGG, AAAYY, ALLAG	NLRC3. NLR family CARD domain-containing protein 3
LPPLL	NR1H2. Oxysterol receptor LXR-β
GVLTE	NR1H4. Bile acid receptor
PIGAG	NR5A2. Nuclear receptor subfamily 5 group A member 2
VAVLY	PACR. Pituitary adenylate cyclase-activating polypeptide type I receptor
YSVLY	PD2R. Prostaglandin D2 receptor
AISSV	PE2R2. Prostaglandin E2 receptor EP2 subtype
TLADA	PTH2R. Parathyroid hormone 2 receptor
AEIRA	PTHR. Parathyroid hormone-related protein
KLQDV	PTHY. Parathyroid hormone
AVRDP	RAMP1. Receptor activity-modifying protein 1
NNTVY	RORA. Nuclear receptor ROR-α
GTITS	RXFP1. Relaxin receptor 1
PDKVF	RXFP2. Relaxin receptor 2
QQFGR, TGIAV	SBNO2. Protein strawberry notch homolog 2
VAVLY	SCTR. Secretin receptor
AGAAA	SECR. Secretin
VAVLY, TSPDV	VIPR2. Vasoactive intestinal polypeptide receptor 2

^a^ Hexa- and heptapeptides composed of overlapping pentapeptides are given in bold. ^b^ Proteins given by Uniprot entry and name. Disease association and references are available at Uniprot, PubMed, and OMIM public databases.

**Table 3:** Immunoreactive SARS-CoV-2 spike gp-derived epitopes containing peptides shared between the SARS-CoV-2 spike gp and anti-inflammatory human proteins.

IEDB ID ^1^	Epitope Sequence ^2^	IEDB ID ^1^	Epitope Sequence ^2^
1069137	aqytsALLAGTITSg	1309574	rSSVLHstqdlflPFFSNvt
1069290	ctlksftvEKGIYqt	1309578	sfiedllfnkvTLADAgfik
1069291	cvadYSVLYnsasfs	1309585	sssgwtAGAAAYYvgylqpr
1069378	ecdiPIGAGICASyq	1309589	sygfqpTNGVGyqpyrvvvl
1071585	nLVRDLpqgFSALEp	1309600	tyvtqqLIRAAEIRAsanla
1072807	skhtpinLVRDLpqg	1309603	vknkcvnfnFNGLTgtgvlt
1073281	tesnkkflpfQQFGRdia	1309604	vlndilsrldKVEAEvqidr
1074838	AEIRAsanlaatk	1309606	vTLADAgfikqygdclgdia
1074847	aphgvVFLHVtyv	1309616	yeqyikwpwyiwLGFIAgli
1074888	flPFFSNvtwfhai	1309621	yskhtpinLVRDLpqgfsal
1075041	rsvasQSIIAytmsl	1310281	aphgvVFLHVtyvpa
1075079	tpinLVRDL	1310282	aqalntlvKQLSSNf
1075094	VLPPLLtdemiaqyt	1310303	caqkFNGLTVLPPLL
1075117	wtAGAAAYYvgy	1310415	FNGLTVLPPLLtdem
1075125	YSVLYnsASFSTfk	1310434	gAISSVlndilsrld
1087359	iPIGAGICASy	1310444	givNNTVYdplqpel
1087679	pikdfggfnFSQILpdps	1310445	gIYQTSnfrvqptes
1087680	pinLVRDLpqgFSALEpl	1310448	gKLQDVvnqnaqaln
1125063	gltVLPPLL	1310487	iginitrfqTLLALh
1309123	khtpinLVRDLpqgf	1310513	itrfqTLLALhrsyl
1309132	nFSQILpdpskpskr	1310551	krisncvadYSVLYn
1309140	tdemiaqytsALLAG	1310593	llfnkvTLADAgfik
1309418	AEIRAsanlaatkmsecvlg	1310611	LPPLLtdemiaqyts
1309444	dAVRDPqTLEILDitpcsfg	1310612	lpqgFSALEplvdlp
1309447	dfggfnFSQILpdpskpskr	1310787	sASFSTfkcygvspt
1309450	dplsetkctlksftvEKGIY	1310803	siiayTMSLGaensv
1309451	dsfkeeldkyfknhTSPDVd	1310825	svasQSIIAyTMSLG
1309461	ehvnnsyecdiPIGAGICAS	1310828	svlynsASFSTfkcy
1309464	esnkkflpfQQFGRdiadtt	1310850	TLEILDitpcsfggv
1309469	fknhTSPDVdlgdisginas	1310852	tlvKQLSSNFGaiss
1309490	iawnsnnldSKVGGnynyly	1310865	trfqTLLALhrsylt
1309522	lppaytnsftrgvyyPDKVF	1310899	vLLPLVssqcvnltt
1309523	lSSNFGAISSVlndilsrld	1313244	nsASFSTfk
1309531	ngltgtGVLTEsnkkflpfq	1313285	pinLVRDLpqgfsal
1309532	ngltVLPPLLtdemiaqyts	1313286	pinLVRDLpqgfwal
1309534	nitrfqTLLALhrsyltpgd	1316945	FSQILpdpskpskrsfie
1309546	pflmdlegkqgnfKNLREfv	1321084	LPPLLtdem
1309558	qfnsaigkiQDSLSstasal	1325128	svasQSIIAy
1309561	qrnfyepqiittdNTFVSGn	1326261	vasQSIIAy
1309567	rdlpqgFSALEplvdlpigi	1328800	yTMSLGaensvay

^1^ Epitopes listed according to the IEDB ID number.^2^ Shared peptides are capitalized.

**Table 4:** Pentapeptides occurring in microbial organisms and common to the SARS-CoV-2 spike gp, SARS-CoV-2 spike gp-derived epitopes, and anti-inflammatory proteins.

Organism	Shared Pentapeptides
*B. pertussis*	AEIRA, AGAAA, AISSV, ALLAG, IRAAE, LEILD, LIRAA, LLPLV, LVRDL, SKVGG
*C. diphtheriae*	AEIRA, AGAAA, ALLAG, AVRDP, LLPLV, TLADA, VLPPL
*C. tetani*	AGAAA, EKGIY, GVLTE, RAAEI, TLADA
*H. influenzae*	AEIRA, AGAAA, AISSV, FNGLT, FSALE, GTITS, GVLTE, IRAAE, KNLRE, KQLSS, KVEAE, LEILD, LGFIA, LIRAA, LLPLV, LPPLL, LVRDL, QDSLS, QLSSN, QSIIA, RAAEI, TLADA, TLEIL, TLLAL, TMSLG, TNGVG, TSPDV, VLPPL
*N. meningitidis*	AEIRA, AGAAA, ALLAG, ASFST, FNGLT, FSALE, LEILD, LGFIA, LPPLL, LVRDL, QDSLS, SKVGG, TLLAL, TMSLG

## Supplementary Materials

The following is available online at https://www.mdpi.com/article/10.3390/antib10040036/s1: Table S1. List of 219 human proteins assembled at random from UniprotKB database by using “anti-inflammatory” as keyword.
