# Supplementary material for: From Anti-SARS-CoV-2 Immune Response to the Cytokine Storm via Molecular Mimicry
Source: Antibodies (Basel). 2021 Sep 24;10(4):36. doi: 10.3390/antib10040036 (PMC8544210; doi:10.3390/antib10040036)
Supplement: Supplementary file 1 [file antibodies-10-00036-s001.zip › Antibodies 2- Supplemental Table S1.pdf]

Table S1. List of 219 human proteins assembled at random from UniprotKB database by using "anti-inflammatory" as keyword. Proteins given by Uniprot entry and name.

5HT4R. 5-hydroxytryptamine receptor 4
5HT6R. 5-hydroxytryptamine receptor 6
5HT7R. 5-hydroxytryptamine receptor 7
AA2AR. Adenosine receptor A2a
AA2BR. Adenosine receptor A2b
ACTHR. Adrenocorticotropic hormone receptor
ADA17. Disintegrin and metalloproteinase domain-containing protein 17
ADCY1. Adenylate cyclase type 1
ADCY2. Adenylate cyclase type 2
ADCY3. Adenylate cyclase type 3
ADCY4. Adenylate cyclase type 4
ADCY5. Adenylate cyclase type 5
ADCY6. Adenylate cyclase type 6
ADCY7. Adenylate cyclase type 7
ADCY8. Adenylate cyclase type 8
ADCY9. Adenylate cyclase type 9
ADIPO. Adiponectin
ADM2. Protein ADM2
ADML. Pro-adrenomedullin
ADRB1. Beta-1 adrenergic receptor
ADRB2. Beta-2 adrenergic receptor
ADRB3. Beta-3 adrenergic receptor
AIM2. Interferon-inducible protein AIM2
AK1C3. Aldo-keto reductase family 1 member C3
AMACR. Alpha-methylacyl-CoA racemase
ANXA1. Annexin A1
ASIC1. Acid-sensing ion channel 1
ASIC2. Acid-sensing ion channel 2
ASIC3. Acid-sensing ion channel 3
C163A. Scavenger receptor cysteine-rich type 1 protein M130
C1QT4. Complement C1q tumor necrosis factor-related protein 4
CALC. Calcitonin
CALCA. Calcitonin gene-related peptide 1
CALCB. Calcitonin gene-related peptide 2
CALCR. Calcitonin receptor
CALRL. Calcitonin gene-related peptide type 1 receptor
CCN3. CCN family member 3
CDK9. Cyclin-dependent kinase 9
CLTR2. Cysteinyl leukotriene receptor 2
COLI. Pro-opiomelanocortin

CP4F2. Cytochrome P450 4F2
CP4F3. Cytochrome P450 4F3
CREB1. Cyclic AMP-responsive element-binding protein 1
CRF. Corticoliberin
CRFR1. Corticotropin-releasing factor receptor 1
CSF1R. Macrophage colony-stimulating factor 1
CXL17. C-X-C motif chemokine 17
DP13B. DCC-interacting protein 13-beta
DRD1. D(1A) dopamine receptor
DRD5. D(1B) dopamine receptor
FFAR1. Free fatty acid receptor 1
FFAR4. Free fatty acid receptor 4
FNDC4. Fibronectin type III domain-containing protein 4
FPR2. N-formyl peptide receptor 2
FSHB. Follitropin subunit beta
FSHR. Follicle-stimulating hormone receptor
FURIN. Furin
GBB1. Guanine nucleotide-binding protein G(I)/G(S)/G(T) subunit beta-1
GBB2. Guanine nucleotide-binding protein G(I)/G(S)/G(T) subunit beta-2
GBB3. Guanine nucleotide-binding protein G(I)/G(S)/G(T) subunit beta-3
GBB4. Guanine nucleotide-binding protein subunit beta-4
GBG1. Guanine nucleotide-binding protein G(T) subunit gamma-T1
GBG10. Guanine nucleotide-binding protein G(I)/G(S)/G(O) subunit gamma-10
GBG11. Guanine nucleotide-binding protein G(I)/G(S)/G(O) subunit gamma-11
GBG12. Guanine nucleotide-binding protein G(I)/G(S)/G(O) subunit gamma-12
GBG13. Guanine nucleotide-binding protein G(I)/G(S)/G(O) subunit gamma-13
GBG2. Guanine nucleotide-binding protein G(I)/G(S)/G(O) subunit gamma-2
GBG3. Guanine nucleotide-binding protein G(I)/G(S)/G(O) subunit gamma-3
GBG4. Guanine nucleotide-binding protein G(I)/G(S)/G(O) subunit gamma-4
GBG5. Guanine nucleotide-binding protein G(I)/G(S)/G(O) subunit gamma-5
GBG7. Guanine nucleotide-binding protein G(I)/G(S)/G(O) subunit gamma-7
GBG8. Guanine nucleotide-binding protein G(I)/G(S)/G(O) subunit gamma-8
GBGT2. Guanine nucleotide-binding protein G(I)/G(S)/G(O) subunit gamma-T2
GDE1. Glycerophosphodiester phosphodiesterase 1
GHRHR. Growth hormone-releasing hormone receptor
GIP. Gastric inhibitory polypeptide
GIPR. Gastric inhibitory polypeptide receptor
GLHA. Glycoprotein hormones alpha chain
GLP1R. Glucagon-like peptide 1 receptor
GLP2R. Glucagon-like peptide 2 receptor
GLUC. Pro-glucagon
GNAI1. Guanine nucleotide-binding protein G(i) subunit alpha-1
GNAI2. Guanine nucleotide-binding protein G(i) subunit alpha-2

GNAI3. Guanine nucleotide-binding protein G(i) subunit alpha-3
GNAS1. Guanine nucleotide-binding protein G(s) subunit alpha isoforms XLas
GNAS2. Guanine nucleotide-binding protein G(s) subunit alpha isoforms short
GNAT3. Guanine nucleotide-binding protein G(t) subunit alpha-3
GNAZ. Guanine nucleotide-binding protein G(z) subunit alpha
GNB5. Guanine nucleotide-binding protein subunit beta-5
GP150. Probable G-protein coupled receptor 150
GP176. G-protein coupled receptor 176
GPBAR. G-protein coupled bile acid receptor 1
GPHA2. Glycoprotein hormone alpha-2
GPHB5. Glycoprotein hormone beta-5
GPR15. G-protein coupled receptor 15
GPR20. G-protein coupled receptor 20
GPR25. Probable G-protein coupled receptor 25
GPR27. Probable G-protein coupled receptor 27
GPR32. Probable G-protein coupled receptor 32
GPR39. G-protein coupled receptor 39
GPR45. Probable G-protein coupled receptor 45
GPR83. Probable G-protein coupled receptor 83
GPR84. G-protein coupled receptor 84
GPS2. G protein pathway suppressor 2
GSTM4. Glutathione S-transferase Mu 4
HMR1. Major histocompatibility complex class I-related gene protein
HRH2. Histamine H2 receptor
I10R1. Interleukin-10 receptor subunit alpha
I36RA. Interleukin-36 receptor antagonist protein
IAPP. Islet amyloid polypeptide
IF16. Gamma-interferon-inducible protein 16
IL10. Interleukin-10
IL27A. Interleukin-27 subunit alpha
IL27B. Interleukin-27 subunit beta
IL37. Interleukin-37
IL6. Interleukin-6
IL6RA. Interleukin-6 receptor subunit alpha
INSL3. Insulin-like 3
ISK5. Serine protease inhibitor Kazal-type 5
KAP0. cAMP-dependent protein kinase type I-alpha regulatory subunit
KAP1. cAMP-dependent protein kinase type I-beta regulatory subunit
KAP2. cAMP-dependent protein kinase type II-alpha regulatory subunit
KAP3. cAMP-dependent protein kinase type II-beta regulatory subunit
KAPCA. cAMP-dependent protein kinase catalytic subunit alpha
KAPCB. cAMP-dependent protein kinase catalytic subunit beta
KAPCG. cAMP-dependent protein kinase catalytic subunit gamma

KEAP1. Kelch-like ECH-associated protein 1
KS6A4. Ribosomal protein S6 kinase alpha-4
KS6A5. Ribosomal protein S6 kinase alpha-5
LKHA4. Leukotriene A-4 hydrolase
LOX12. Polyunsaturated fatty acid lipoxygenase ALOX12
LOX15. Polyunsaturated fatty acid lipoxygenase ALOX15
LOX5. Polyunsaturated fatty acid 5-lipoxygenase
LSHB. Lutropin subunit beta
LSHR. Lutropin-choriogonadotropic hormone receptor
LTC4S. Leukotriene C4 synthase
LX15B. Polyunsaturated fatty acid lipoxygenase ALOX15B
MC3R. Melanocortin receptor 3
MC4R. Melanocortin receptor 4
MC5R. Melanocortin receptor 5
METRL. Meteorin-like protein
MFS10. Major facilitator superfamily domain-containing protein 10
MIF. Macrophage migration inhibitory factor
MK14. Mitogen-activated protein kinase 14
MSHR. Melanocyte-stimulating hormone receptor
MXRA5. Matrix-remodeling-associated protein 5
MY18A. Unconventional myosin-XVIIIa
MYH9. Myosin-9
NAL10. NACHT, LRR and PYD domains-containing protein 10
NEU2. Vasopressin-neurophysin 2-copeptin
NF2L2. Nuclear factor erythroid 2-related factor 2
NFKB1. Nuclear factor NF-kappa-B p105 subunit
NLRC3. NLR family CARD domain-containing protein 3
NPS. Neuropeptide S
NPSR1. Neuropeptide S receptor
NR1H2. Oxysterols receptor LXR-beta
NR1H4. Bile acid receptor
NR5A2. Nuclear receptor subfamily 5 group A member 2
P2Y11. P2Y purinoceptor 11
PA2GD. Group IID secretory phospholipase A2
PACA. Pituitary adenylate cyclase-activating polypeptide
PACR. Pituitary adenylate cyclase-activating polypeptide type I receptor
PAFA. Platelet-activating factor acetylhydrolase
PD2R. Prostaglandin D2 receptor
PDE4B. cAMP-specific 3',5'-cyclic phosphodiesterase 4B
PE2R2. Prostaglandin E2 receptor EP2 subtype
PE2R4. Prostaglandin E2 receptor EP4 subtype
PGDH. 15-hydroxyprostaglandin dehydrogenase
PGH1. Prostaglandin G/H synthase 1

PGH2. Prostaglandin G/H synthase 2
PI2R. Prostacyclin receptor
PK3CG. Phosphatidylinositol 4,5-bisphosphate 3-kinase catalytic subunit gamma isoform
PLK2. Serine/threonine-protein kinase PLK2
PRKX. cAMP-dependent protein kinase catalytic subunit PRKX
PTGR1. Prostaglandin reductase 1
PTH1R. Parathyroid hormone/parathyroid hormone-related peptide receptor
PTH2R. Parathyroid hormone 2 receptor
PTHR. Parathyroid hormone-related protein
PTHY. Parathyroid hormone
PXL2A. Peroxiredoxin-like 2A
RAMP1. Receptor activity-modifying protein 1
RAMP2. Receptor activity-modifying protein 2
RAMP3. Receptor activity-modifying protein 3
RARR2. Retinoic acid receptor responder protein 2
REL2. Prorelaxin H2
REL3. Relaxin-3
RHDF2. Inactive rhomboid protein 2
RORA. Nuclear receptor ROR-alpha
RXFP1. Relaxin receptor 1
RXFP2. Relaxin receptor 2
RXRA. Retinoic acid receptor RXR-alpha
S10A8. Protein S100-A8
SBNO2. Protein strawberry notch homolog 2
SC5A8. Sodium-coupled monocarboxylate transporter 1
SCTR. Secretin receptor
SECR. Secretin
SG3A2. Secretoglobin family 3A member 2
SLIB. Somatoliberin
SLPI. Antileukoproteinase
T22D3. TSC22 domain family protein 3
TAAR1. Trace amine-associated receptor 1
TAAR2. Trace amine-associated receptor 2
TAAR3. Putative trace amine-associated receptor 3
TAAR5. Trace amine-associated receptor 5
TAAR6. Trace amine-associated receptor 6
TAAR8. Trace amine-associated receptor 8
TAAR9. Trace amine-associated receptor 9
TIE2. Angiopoietin-1 receptor
TIP39. Tuberoinfundibular peptide of 39 residues
TNIP1. TNFAIP3-interacting protein 1
TREM2. Triggering receptor expressed on myeloid cells 2
TSHB. Thyrotropin subunit beta

TSHR. Thyrotropin receptor
TTP. mRNA decay activator protein ZFP36
V2R. Vasopressin V2 receptor
VIP. VIP peptides
VIPR1. Vasoactive intestinal polypeptide receptor 1
VIPR2. Vasoactive intestinal polypeptide receptor 2
ZC12A. Endoribonuclease ZC3H12A
